# Combinatorial Sec pathway analysis for improved heterologous protein secretion in *Bacillus subtilis*: identification of bottlenecks by systematic gene overexpression

**DOI:** 10.1186/s12934-015-0282-9

**Published:** 2015-06-26

**Authors:** Jingqi Chen, Gang Fu, Yuanming Gai, Ping Zheng, Dawei Zhang, Jianping Wen

**Affiliations:** Department of Biological Engineering, School of Chemical Engineering and Technology, Tianjin University, Tianjin, 300072 People’s Republic of China; Tianjin Institute of Industrial Biotechnology, Chinese Academy of Sciences, Tianjin, 300308 People’s Republic of China; Key Laboratory of Systems Microbial Biotechnology, Chinese Academy of Sciences, Tianjin, 300308 People’s Republic of China; National Engineering Laboratory for Industrial Enzymes, Tianjin, 300308 People’s Republic of China

**Keywords:** *Bacillus subtilis*, Bottleneck, Heterologous protein, Overexpression, Sec pathway

## Abstract

**Background:**

Secretory expression of valuable proteins by *B. subtilis* and its related species has attracted intensive work over the past three decades. Although very high yields can be achieved with homologous proteins, production of heterologous proteins by *B. subtilis* is unfortunately not the straight forward. The Sec pathway is the major route for protein secretion in *B. subtilis*. Therefore, the aim of this work was to identify the bottlenecks of the Sec pathway and improve the secretion of heterologous proteins by molecular genetic techniques.

**Results:**

Two α-amylases (AmyL and AmyS) both under the control of the P_*HpaII*_ promoter and equipped with their native signal peptides SP_*amyl*_ and SP_*amyS*_ were successfully secreted with significantly different expression levels. To improve the secretion efficiency, 23 main genes or gene operons involved in or closely related to the Sec pathway were overexpressed singly by increasing an additional copy on the chromosome, and the overexpression of *prsA* enhanced the production of α-amylases (AmyL and AmyS) by 3.2- and 5.5-fold, respectively. With the induction by xylose of different concentrations, *prsA* overexpression level was optimized and the secretion efficiency of α-amylase was further improved. Moreover, combinatorial overexpression of *prsA* and nine screened genes or gene operons, respectively, was performed, and the overexpression of *prsA* combined with partial *dnaK* operon improved the α-amylase activity of AmyL and AmyS by 160 and 173%, respectively, compared with the overexpression of *prsA* singly. Finally, the performance of the recombinant *B. subtilis* 1A237 was evaluated with the fed-batch fermentation in 7.5 L fermentor, and the level of secreted AmyL and AmyS reached 1,352 and 2,300 U/mL with the productivity of 16.1 U/mL h and 27.4 U/mL h, respectively.

**Conclusions:**

Our systematic gene overexpression approach was designed to investigate the bottleneck of Sec pathway in *B. subtilis*. The deficiency of PrsA lipoprotein and chaperones of DnaK series was main rate-limiting factors for heterologous proteins secretion. Systematic and deep insight into how components of Sec pathway interact with each other may be the key to improving the yield of heterologous proteins thoroughly.

**Electronic supplementary material:**

The online version of this article (doi:10.1186/s12934-015-0282-9) contains supplementary material, which is available to authorized users.

## Backgroud

The Gram-positive bacterium *Bacillus subtilis* is widely known for its capacity to produce and secrete large amounts of industrially relevant proteins, mostly endogenous enzymes like amylases, proteases and lipases. In particular, the ability of *B. subtilis* to secrete proteinaceous products into the growth medium greatly facilitates downstream processing. The use of *B. subtilis* also has many other advantages, such as its GRAS (generally regarded as safe) status [1, 2], high amenability for genetic engineering [3], no pronounced codon bias [4] and the easy and inexpensive culturing methods and large-scale fermentation. Thus, scientists pay more attention to this organism with the aim to the commercial exploitation of *B. subtilis* as major “cell factories” for secreted heterologous proteins of interest.

In *B. subtilis*, the major route of protein transport across the cytoplasmic membrane is the general secretion (Sec) pathway [5, 6]. This involves four types of secretory machinery components: (1) Signal recognition particle (SRP). SRP, a highly conserved and essential ribonucleoprotein complex, is in charge of recognizing the SP sequence of a nascent chain and targeting it to the membrane. *B. subtilis* SRP consists of a small cytoplasmic RNA (scRNA) [7], a GTPase Ffh [8] and two molecules of Hbsu protein [9]. Together they form the SRP complex, which is recognized by the membrane bound SRP receptor Ftsy. Ftsy targets the SRP complex to the translocase complex [10]. In addition, it has been discovered that CsaA which seems to serve as a SecB homologue [11], can directly interact with SecA and precursor proteins to influence the secretory efficiency. (2) Translocase complex. In *B. subtilis*, the Sec translocases consist of SecA (motor protein) [12, 13], a heterotrimeric SecYEG complex (pore) [14–16], SecDF (chimeric protein) [17], YrbF (YajC homolgue) [18], and YidC homologues (SpoIIIJ and YqjG) [19]. The SecY, SecE and SecG proteins form the SecYEG complex. In general, the limited secretory efficiency is attributed to the insufficient capacity of the transport machinery. (3) Signal peptidases. In *B. subtilis*, five *sip* genes (*sipT*, *sipS*, *sipU*, *sipV* and *sipW*) for type I SPases have been identified. SipS and SipT are key to preprotein processing, while SipU, SipV and SipW appear to play minor roles in protein secretion. (4) Chaperones. *B. subtilis* has two types of molecular chaperones, intracellular and extracytoplasmic molecular chaperones. GroE and DnaK series are intracellular molecular chaperones mediating protein folding, minimizing aggregation and maintaining preproteins in translocation-competent conformations. The genes for these chaperones are organized in two operons, the *groESL* operon (*groES*-*groEL*) and the partial *dnaK* operon (*grpE*-*dnaK*-*dnaJ*). The extracytoplasmic folding factor PrsA is a well-defined lipoprotein required for subsequent folding of the mature protein into a stable and active conformation.

Although very high yields can be achieved with homologous proteins [20], production of heterologous proteins by *B. subtilis* is quite problematic [21]. In theory every step in the process of protein production and translocation can be a bottleneck causing reduced yields. As a result, it is imperative to explore the secretory process and engineer the secretory machinery components to improve the secretory efficiency. Recently, the overexpression of Ftsy and/or SRP components to improve protein secretion is described in a patent [22], as is the use of *Pseudomonas glumea* chaperones to improve the stability of the secretory proteins [23]. Diao constructed an artificial protein targeting pathway by co-expressing SecB (*E. coli*) and a *B. subtilis* hybrid SecA (the C-terminal 32 amino acids were replaced by the corresponding fragment of SecA from *E. coli*), and successfully increased the secretion of both MalE11 and PhoA [5]. Kakeshita deleted the C-terminal region of SecA, and the extracellular production of heterologous proteins was enhanced successfully in *B. subtilis* [13]. Mulder constructed an artificial *secYEG* operon to optimize their expression and substantially increased the secretory production of α-amylase [14]. Nouaille complemented the *Lactococcus lactis* Sec machinery with SecDF from *B. subtilis* and the secretion of *Staphylococcal* Nuclease was obviously improved [24]. Malten had successfully increased the secretory production of recombinant protein by overexpressing the type I SPase in *B. megaterium* [25]. Wu reduced the formation of inclusion bodies and increased the secretory production yield of single chain antibody (SCA) in *B. subtilis* WB600, by coproducing GroE operon, Dnak operon and PrsA [26]. In many cases, overexpression of secreted proteins can cause jamming of membrane because of the shortage of Sec pathway components. The researches stated above notably suggest that modification or overexpression of Sec pathway components is an efficient approach for improving the secretory capacity of *B. subtilis*.

Although some specific components involved in Sec pathway were investigated, systematic studies of the effect of every component on heterologous protein secretion in *B. subtilis* are few. In this work, we described a systematic program to engineer the secretory machinery components to improve the secretory efficiency, namely: (1) effect of overexpression of every component involved in Sec pathway on heterologous protein secretion and identification of main bottlenecks in the secretory process in *B. subtilis*; (2) optimization of PrsA lipoprotein overexpression on expression level, providing the most efficient folding. (3) effect of PrsA overexpression in combination with other components screened out, respectively, on heterologous protein secretion. To reveal the bottlenecks of protein secretion process more convincingly, we chose two heterologous α-amylases, AmyL (α-amylase from *Bacillus licheniformis*) and AmyS (α-amylase from *Geobacillus stearothermophilus*), as target proteins, which were secreted in distinctly different levels by *B. subtilis*. In this manner, we found that the deficiency of PrsA lipoprotein and chaperones of Dnak series is the most critical rate-limiting step in protein secretion. By overexpression of both intracellular and extracellular chaperones simultaneously, the enzyme activity and production of AmyL and AmyS were increased to approximately 9- and 12-fold, correspondingly. Furthermore, the production of AmyL and AmyS by *B. subtilis* was conducted in 7.5 L fermentor with fed-batch strategy, respectively.

## Results

### Expression of recombinant α-amylase AmyL and AmyS in *B. subtilis*

**Figure 1:**
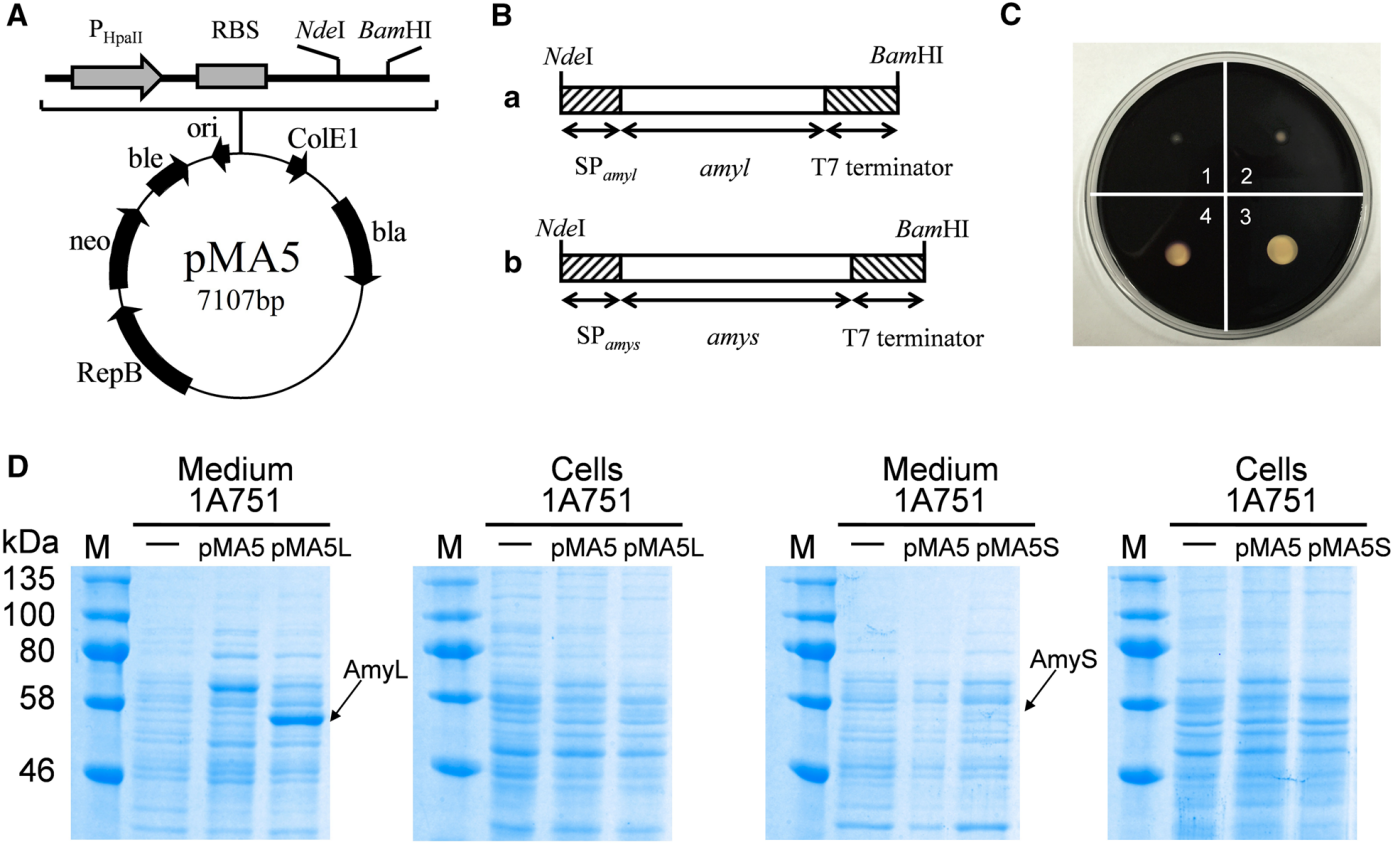
Expression and secretion of α-amylase (AmyL and AmyS) in *B. subtilis* 1A751. **A** Vector map of *E. coli*/*B. subtilis* shuttle plasmid pMA5. P_*HpaII*_, a widely used promoter from *Staphylococcus aureus*; RBS, ribosome binding site; ColE1, origin of replication for *E. coli*; *bla*, ampicillin resistance; RepB, origin of replication for *B. subtilis*; *neo*, kanamycin resistance. **B** The gene fragments of α-amylase (AmyL and AmyS). Fragment a: AmyL encoding gene containing its native signal peptide SP_*amyl*_ plus T7 terminator; fragment b: AmyS encoding gene containing its native signal peptide SP_*amys*_ plus T7 terminator. **C** Starch hydrolysis of *B. subtilis* 1A751 (1), 1A751 (pMA5) (2), 1A751 (pMA5L) (3), 1A751 (pMA5S) (4). Strains were grown on LB agar medium containing 1% starch at 37°C overnight. Flood the surface of the plate with 5 mL Gram’s iodine stain. **D** SDS-PAGE analysis of expression of α-amylase (AmyL and AmyS) in medium and cell fractions by *B. subtilis* at incubation of 72 h. *Lane M* molecular weight marker.

DNA fragment (Figure 1B) coding for thermostable α-amylase AmyL or AmyS with their own signal peptide was obtained from *B. licheniformis* CICC 10181 or *G. stearothermophilus* ATCC 31195 by PCR and inserted into the *E. coli*/*B. subtilis* shuttle plasmid pMA5 (Figure 1A), under the control of the strong and constitutive promoter P_*HpaII*_ and upstream of the T7 terminator from *E. coli*. The obtained target plasmids pMA5L and pMA5S were transformed into *B. subtilis* 1A751, respectively, resulting into recombinant strains by selection at 37°C on the LB agar plates containing 50 µg/mL kanamycin and 1% soluble starch. The transformants with transparent rings around colonies were positive (Figure 1C). The insertion of AmyL or AmyS gene in the transformants was confirmed by PCR. The *B. subtilis* 1A751 transformed with plasmid pMA5 was used as the control. The colonies were grown in 30 mL of SR medium at 37°C for 72 h in 250 mL shaker flasks. With the direction of signal peptide, mature AmyL and AmyS were released into extracellular medium. The α-amylase activity of AmyL and AmyS in the culture supernatant was 90 and 111 U/mL, respectively. The α-amylase activity in the culture supernatant of the control strain was not detected under the same culture conditions. Meanwhile, SDS-PAGE analysis showed that a distinct and a slight bands with a molecular mass of about 55-kDa which is in good agreement with the deduced value were observed in the medium fraction of AmyL producer and AmyS producer, respectively (Figure 1D). However, there was nearly no accumulation of AmyL and AmyS in the cell fraction. The results indicate that the SP_*amyl*_ and SP_*amys*_ both have relatively high secretion efficiency. In addition, we can know that AmyL and AmyS are expressed in significantly different levels.

### Development of an integration-vector system for co-overexpression of two different target genes

To successfully increase a copy of the target genes or gene operons involved in Sec pathway on the chromosome in *B. subtilis*, we constructed an integration vector pDDXG (Additional file 1: Figure S1a), derivative from the vector pDD which contains *amyE* front and back regions for integration into the *B. subtilis* chromosome at *amyE* locus via double crossing-over. Firstly, we constructed the plasmid pDDX by inserting the strong and inducible promoter P_*xylA*_ into the vector pDD. As an upgrade, the integration vector pDDXG which could be used to overexpress two different genes simultaneously was constructed by inserting a strong and constitutive promoter P_*grac*_ into the vector pDDX downstream of the promoter P_*xylA*_. To verify that the constructed pDDXG vector could fulfill the requirement, we constructed pDDXBG containing *bgaB* gene under the control of the promoter P_*xylA*_ and pDDXGB containing *bgaB* gene under the control of the promoter P_*grac*_, and then these two *Ahd*I-linearized plasmids were integrated into the chromosome of *B. subtilis* 1A751 via double crossing-over, respectively. β-galactosidase activity was detected in both recombinant 1A751 (P_*xylA*_-*bgaB*) after induction by 2.0% xylose and 1A751 (P_*grac*_-*bgaB*) without any induction (data not shown). This showed that pDDXG could be successfully used to overexpress one or two genes by integrating the target gene(s) into the chromosome.

### Effect of overexpression of single gene involved in Sec pathway on α-amylase secretion

**Figure 2:**
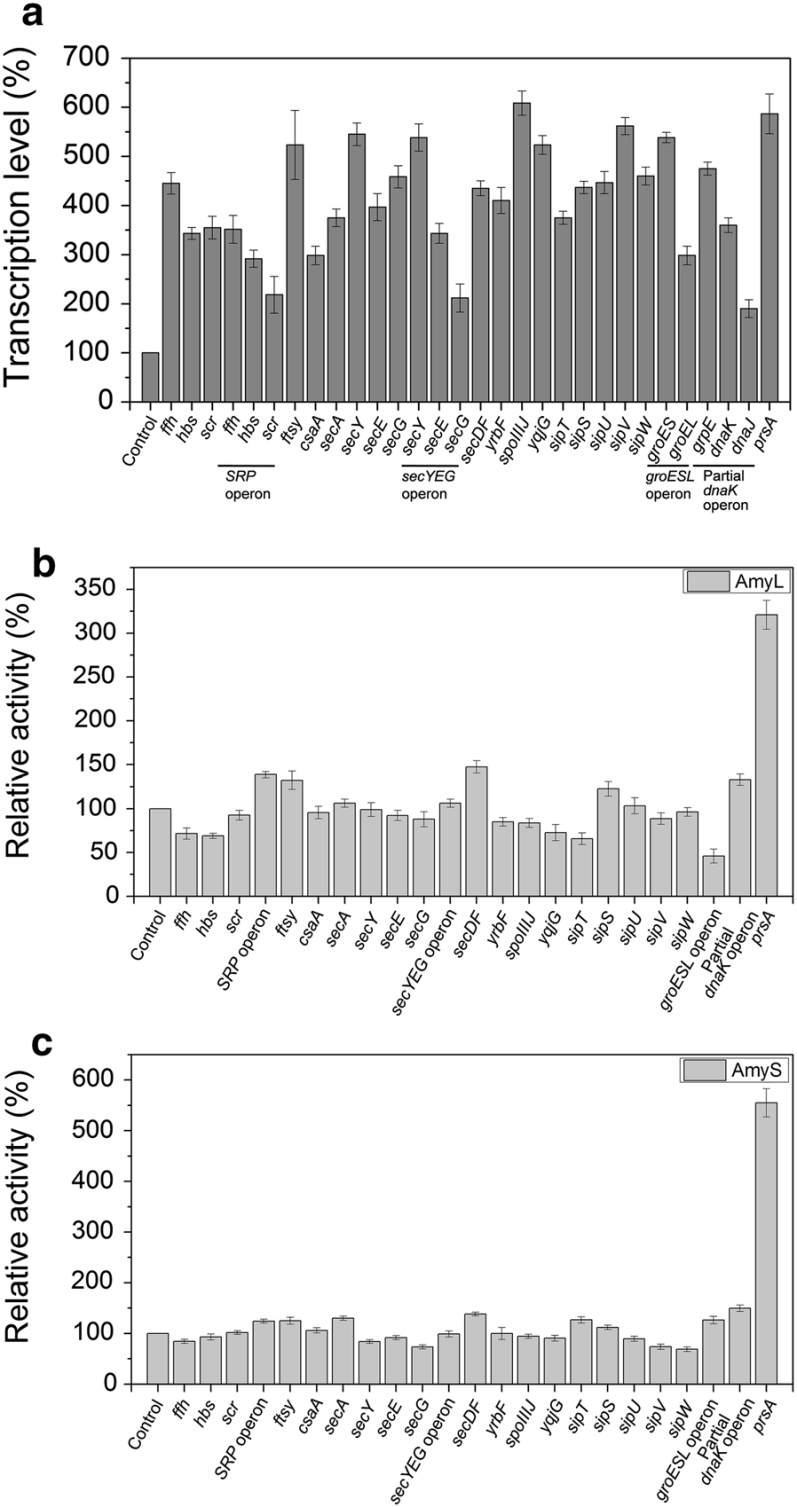
Overexpression of single component involved in Sec pathway in *B. subtilis*. **a** The transcriptional levels of 23 genes or gene operons in 23 corresponding recombinant strains, respectively. Column Control represents the transcriptional levels of the 23 genes or gene operons in 1A751 (all regarded as 100%). **b** Effect of overexpression of single gene or gene operon on AmyL secretion. Column Control is the α-amylase activity of 1A751 (pMA5L), regarded as 100%. **c** Effect of overexpression of single gene or gene operon on AmyS secretion. Column Control is the α-amylase activity of 1A751 (pMA5S), served as 100%. The samples were collected at incubation of 72 h in 250 mL shake-flask and centrifuged at 10,000×*g* and 4°C for 10 min. After extraction, the supernatant was used for analysis of α-amylase activity. The α-amylase activity was converted to percent increase referring to the respective control strains. Data represent the mean of three parallel experiments, and *error bars* represent standard error. Specifically, the genes or gene operons *ffh*, *hbs*, *scr*, *SRP*, *ftsy*, *csaA*, *secA*, *secY*, *secE*, *secG*, *secYEG*, *secDF*, *yrbF*, *spoIIIJ*, *yqjG*, *sipT*, *sipS*, *sipU*, *sipV*, *sipW*, *groESL* operon, partial *dnaK* operon and *prsA* in **a**, **b** and **c** corresponded to strains 1A01, 1A02, 1A03, 1A04, 1A05, 1A06, 1A07, 1A08, 1A09, 1A10, 1A11, 1A12, 1A13, 1A14, 1A15, 1A16, 1A17, 1A18, 1A19, 1A20, 1A21, 1A22 and 1A23, respectively.

Overexpression of one or more secretory machinery components has been shown in a number of studies to assist the secretion and folding of several recombinant proteins [14, 25, 27, 28]. To systematically investigate the impact of increased expression of the secretory machinery components on heterologous protein secretion in *B. subtilis*, 23 recombinant strains, 1A01 (*ffh*), 1A02 (*hbs*), 1A03 (*scr*), 1A04 (*SRP*), 1A05 (*ftsy*), 1A06 (*csaA*), 1A07 (*secA*), 1A08 (*secY*), 1A09 (*secE*), 1A10 (*secG*), 1A11 (*secYEG*), 1A12 (*secDF*), 1A13 (*yrbF*), 1A14 (*spoIIIJ*), 1A15 (*yqjG*), 1A16 (*sipT*), 1A17 (*sipS*), 1A18 (*sipU*), 1A19 (*sipV*), 1A20 (*sipW*), 1A21 (*groESL* operon), 1A22 (partial *dnaK* operon) and 1A23 (*prsA*) (Table 1), were constructed by transforming different *Ahd*I-linearized integration vectors (Table 2) containing corresponding genes or gene operons into *B. subtilis* 1A751. The growth of 23 recombinant strains in SR medium was similar to that of the parental strains 1A751. By real-time quantitative PCR, the transcriptional levels of the genes or gene operons involved in Sec pathway in corresponding recombinant strains were examined in the presence of 2.0% xylose so as to induce target genes expression. The respective mRNA levels of the 23 genes or gene operons in corresponding strains were several fold higher than that in 1A751 (Figure 2a).

**Table 1:** Strains used in this study

Strains	Genotype and/or relevant characteristic(s)	Source
*E. coli* DH5α	F^−^∆*lac*U169(Ø80d *lac*Z∆M15) *sup*E44 *hsd*R17 *rec*A1 *gyr*A96 *end*A1 *thi*-1 *rel*A1	Invitrogen
*B. licheniformis* CICC 10181	Wild-type *B. licheniformis*, *amyl* gene	CICC
*G. stearothermophilus* ATCC 31195	Wild-type *G. stearothermophilus, amys* gene	ATCC
*B. subtilis* 1A751	*egl*S∆102 *bgl*T/*bgl*S∆EV *apr*E *npr*E *his*	BGSC
1AXBG	1A751 with integration of pDDXBG (*amyE*::P_*xylA*_-*bgaB*); Cm^r^	This work
1AXGB	1A751 with integration of pDDXGB (*amyE*::P_*grac*_-*bgaB*); Cm^r^	This work
1A01	1A751 with integration of pDD01 (*amyE*::P_*xylA*_-*ffh*); Cm^r^	This work
1A02	1A751 with integration of pDD02 (*amyE*::P_*xylA*_-*hbs*); Cm^r^	This work
1A03	1A751 with integration of pDD03 (*amyE*::P_*xylA*_-*scr*); Cm^r^	This work
1A04	1A751 with integration of pDD04 (*amyE*::P_*xylA*_-*SRP*); Cm^r^	This work
1A05	1A751 with integration of pDD05 (*amyE*::P_*xylA*_-*ftsy*); Cm^r^	This work
1A06	1A751 with integration of pDD06 (*amyE*::P_*xylA*_-*csaA*); Cm^r^	This work
1A07	1A751 with integration of pDD07 (*amyE*::P_*xylA*_-*secA*); Cm^r^	This work
1A08	1A751 with integration of pDD08 (*amyE*::P_*xylA*_-*secY*); Cm^r^	This work
1A09	1A751 with integration of pDD09 (*amyE*::P_*xylA*_-*secE*); Cm^r^	This work
1A10	1A751 with integration of pDD10 (*amyE*::P_*xylA*_-*secG*); Cm^r^	This work
1A11	1A751 with integration of pDD11 (*amyE*::P_*xylA*_-*secYEG*); Cm^r^	This work
1A12	1A751 with integration of pDD12 (*amyE*::P_*xylA*_-*secDF*); Cm^r^	This work
1A13	1A751 with integration of pDD13 (*amyE*::P_*xylA*_-*yrbF*); Cm^r^	This work
1A14	1A751 with integration of pDD14 (*amyE*::P_*xylA*_-*spoIIIJ*); Cm^r^	This work
1A15	1A751 with integration of pDD15 (*amyE*::P_*xylA*_-*yqjG*); Cm^r^	This work
1A16	1A751 with integration of pDD16 (*amyE*::P_*xylA*_-*sipT*); Cm^r^	This work
1A17	1A751 with integration of pDD17 (*amyE*::P_*xylA*_-*sipS*); Cm^r^	This work
1A18	1A751 with integration of pDD18 (*amyE*::P_*xylA*_-*sipU*); Cm^r^	This work
1A19	1A751 with integration of pDD19 (*amyE*::P_*xylA*_-*sipV*); Cm^r^	This work
1A20	1A751 with integration of pDD20 (*amyE*::P_*xylA*_-*sipW*); Cm^r^	This work
1A21	1A751 with integration of pDD21 (*amyE*::P_*xylA*_-*groESL* operon); Cm^r^	This work
1A22	1A751 with integration of pDD22 (*amyE*::P_*xylA*_-partial *dnaK* operon); Cm^r^	This work
1A23	1A751 with integration of pDD23 (*amyE*::P_*xylA*_-*prsA*); Cm^r^	This work
1A231	1A751 with integration of pDD231 (*amyE*::P_*xylA*_-*prsA*, P_*grac*_-*SRP*); Cm^r^	This work
1A232	1A751 with integration of pDD232 (*amyE*::P_*xylA*_-*prsA*, P_*grac*_-*ftsy*); Cm^r^	This work
1A233	1A751 with integration of pDD233 (*amyE*::P_*xylA*_-*prsA*, P_*grac*_-*secA*); Cm^r^	This work
1A234	1A751 with integration of pDD234 (*amyE*::P_*xylA*_-*prsA*, P_*grac*_-*secYEG*); Cm^r^	This work
1A235	1A751 with integration of pDD235 (*amyE*::P_*xylA*_-*prsA*, P_*grac*_-*secDF*); Cm^r^	This work
1A236	1A751 with integration of pDD236 (*amyE*::P_*xylA*_-*prsA*, P_*grac*_-*groESL* operon); Cm^r^	This work
1A237	1A751 with integration of pDD237 (*amyE*::P_*xylA*_-*prsA*, P_*grac*_-partial *dnaK* opeon); Cm^r^	This work
1A238	1A751 with integration of pDD238 (*amyE*::P_*xylA*_-*prsA*, P_*grac*_-*sipT*); Cm^r^	This work
1A239	1A751 with integration of pDD239 (*amyE*::P_*xylA*_-*prsA*, P_*grac*_-*sipS*); Cm^r^	This work

*CICC* China Center of Industrial Culture Collection (http://www.chinacicc.org), *ATCC* American Type Culture Collection, *BGSC* *Bacillus* Genetic Stock Center, USA.

**Table 2:** Plasmids used in this study

Plasmids	Genotype and/or relevant characteristic(s)	Source
pET28-a(+)	*E. coli* expression plasmid, T7 promoter, T7 terminator, Kan^r^	Lab stock
pET28L	pET28-a(+) derivative, *amyl*	This work
pET28S	pET28-a(+) derivative, *amys*	This work
pMA5	*E. coli/B. subtilis* shuttle vector, P_*HpaII*_, Ap^r^, Km^r^	BGSC
pMA5L	pMA5 derivative, SP_*amyl*_, *amyl*, T4 terminator	This work
pMA5S	pMA5 derivative, SP_*amys*_, *amys*, T4 terminator	This work
pHCMC04	P_*xylA*_, Ap^r^, Cm^r^	Lab stock
pHT43	P_*grac*_, SP_*amyQ*_, Ap^r^, Cm^r^	Lab stock
pDD	Integration vector, pDL derivative, Ap^r^, Cm^r^	This work
pDDX	pDD derivative, P_*xylA*_	This work
pDDXG	pDDX derivative, P_*xylA*_, P_*grac*_	This work
pDDXBG	pDDXG derivative, *bgaB*	This work
pDDXGB	pDDXG derivative, *bgaB*	This work
pDD01	pDDXG derivative, *ffh*	This work
pDD02	pDDXG derivative, *hbs*	This work
pDD03	pDDXG derivative, *scr*	This work
pDD04	pDDXG derivative, *SRP*	This work
pDD05	pDDXG derivative, *ftsy*	This work
pDD06	pDDXG derivative, *csaA*	This work
pDD07	pDDXG derivative, *secA*	This work
pDD08	pDDXG derivative, *secY*	This work
pDD09	pDDXG derivative, *secE*	This work
pDD10	pDDXG derivative, *secG*	This work
pDD11	pDDXG derivative, *secYEG*	This work
pDD12	pDDXG derivative, *secDF*	This work
pDD13	pDDXG derivative, *yrbF*	This work
pDD14	pDDXG derivative, *spoIIIJ*	This work
pDD15	pDDXG derivative, *yqjG*	This work
pDD16	pDDXG derivative, *sipT*	This work
pDD17	pDDXG derivative, *sipS*	This work
pDD18	pDDXG derivative, *sipU*	This work
pDD19	pDDXG derivative, *sipV*	This work
pDD20	pDDXG derivative, *sipW*	This work
pDD21	pDDXG derivative, *groESL* operon	This work
pDD22	pDDXG derivative, partial *dnaK* operon	This work
pDD23	pDDXG derivative, *prsA*	This work
pDD231	pDD23 derivative, *prsA*, *SRP*	This work
pDD232	pDD23 derivative, *prsA*, *ftsy*	This work
pDD233	pDD23 derivative, *prsA*, *secA*	This work
pDD234	pDD23 derivative, *prsA*, *secYEG*	This work
pDD235	pDD23 derivative, *prsA*, *secDF*	This work
pDD236	pDD23 derivative, *prsA*, *groESL operon*	This work
pDD237	pDD23 derivative, *prsA*, partial *dnaK operon*	This work
pDD238	pDD23 derivative, *prsA*, *sipT*	This work
pDD239	pDD23 derivative, *prsA*, *sipS*	This work

With construction of the 23 recombinant strains above, the effect of single gene involved in Sec pathway on the production and secretion of heterologous protein was studied systematically. The expression plasmids pMA5L and pMA5S were transformed into the 23 recombinant strains, respectively. The obtained strains were grown in liquid SR medium in the presence of 2.0% xylose, and the α-amylase activity of AmyL and AmyS in medium of the corresponding recombinant strains was measured compared with the parental strains 1A751 at 72 h. For AmyL production (Figure 2b), overexpression of *prsA* resulted in a significant increase (3.2-fold) of the α-amylase activity, and overexpression of *secDF*, *SRP*, partial *dnaK* operon, *ftsy*, *sipS* and *secYEG* resulted in moderate or marginal increase (147, 138, 133, 132, 122 and 108%, respectively; *t* test, all P < 0.05) of the α-amylase activity. However, with the overexpression of other genes, the α-amylase activity was not improved and even reduced. As to AmyS production (Figure 2c), more remarkable improvement (5.5-fold) of the α-amylase activity was observed when *prsA* was overexpressed, and slight enhancements (149, 138, 130, 127, 126, 125, 124 and 112%, respectively; *t* test, all P < 0.05) were obtained when partial *dnaK* operon, *secDF*, *secA*, *sipT*, *groESL* operon, *ftsy*, *SRP* and *sipS* were overexpressed. Similarly, with the overexpression of other genes, the α-amylase activity was also not improved and even reduced. These findings suggest that overexpression of *prsA*, *secDF*, *SRP*, partial *dnaK* operon, *ftsy*, *sipS*, *secYEG*, *secA* or *sipT*, more or less, may enhance the production of α-amylase in *B. subtilis*. Above all, *prsA* is the most vital rate-limiting factor for α-amylase production and secretion in the Sec pathway.

### Optimization of PrsA overexpression level in *B. subtilis*

PrsA is a lipoprotein that consists of a 33-kDa lysine-rich protein part and the N-terminal cysteine with a thiol-linked diacylglycerol anchoring the protein to the outer leaflet of the cytoplasmic membrane [29, 30]. The PrsA lipoprotein is crucial for efficient secretion of a number of exoproteins. Researches show that there is a linear correlation between the number of cellular PrsA molecules and the number of secreted AmyQ molecules over a wide range of *prsA* and *amyQ* expression levels [31]. Meanwhile, our result stated above suggests that PrsA expression level is rate limiting in the secretion of AmyL and AmyS. To further investigate the relationship between α-amylase (AmyL and AmyS) secretion and PrsA expression and enhance the production and secretion of AmyL and AmyS, PrsA overexpression level was optimized.

**Figure 3:**
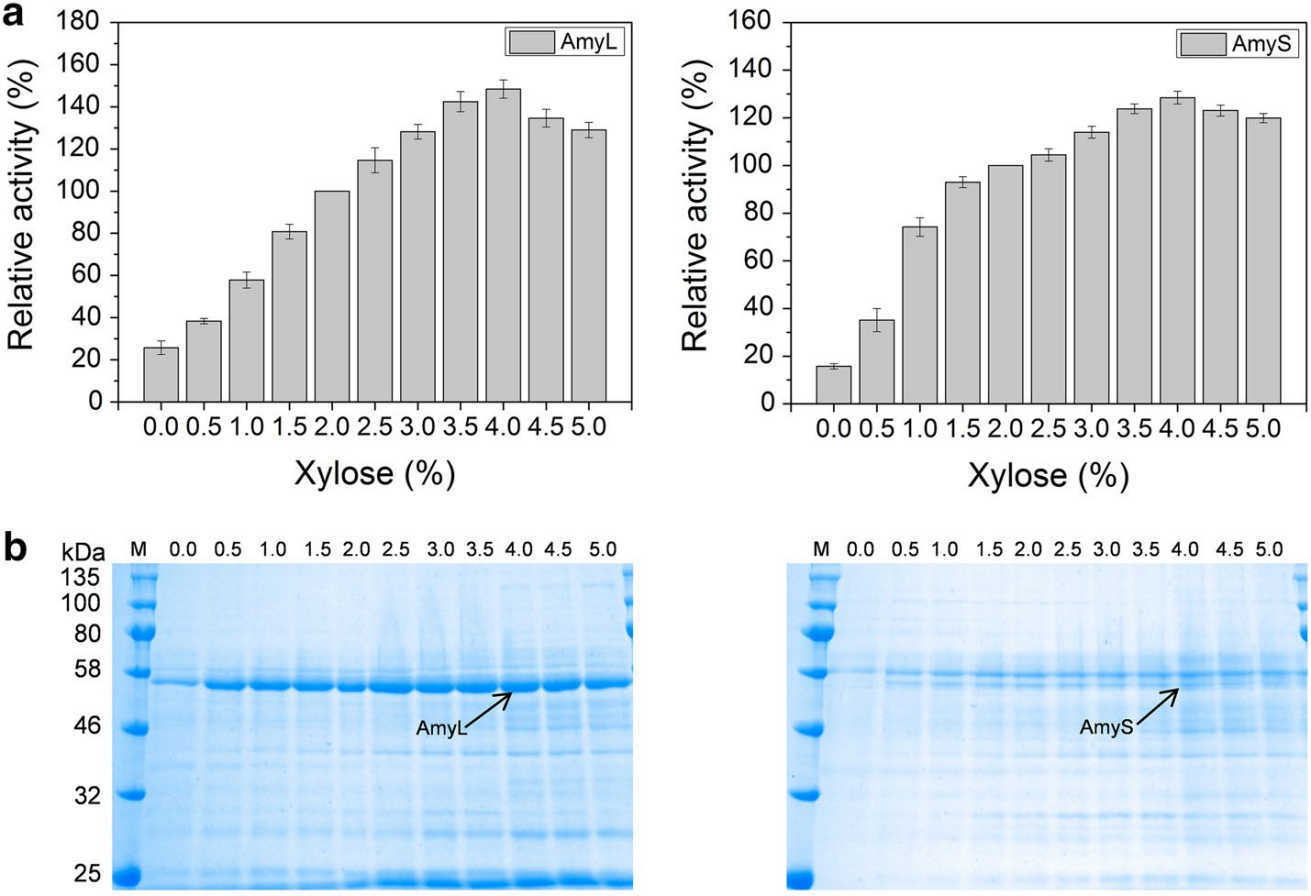
Optimization of PrsA overexpression level in *B. subtilis*. 1A23 (pMA5L) and 1A23 (pMA5S) were grown in SR medium supplemented with xylose of different concentrations [0, 0.5, 1.0, 1.5, 2.0, 2.5, 3.0, 3.5, 4.0, 4.5 and 5.0 % (w/v)]. **a** Analysis of α-amylase (AmyL and AmyS) activity in the supernatant at incubation of 72 h. The α-amylase activity was converted to percent increase referring to 2.0% xylose. **b** SDS-PAGE analysis of α-amylase (AmyL and AmyS) distribution in culture supernatants.

The recombinant strains 1A23 (pMA5L) and 1A23 (pMA5S) secreted constitutively AmyL and AmyS, respectively, and their additional *prsA* has been placed under the control of the promoter P_*xylA*_ at *amyE* locus in chromosome (see Table 1 and “Methods”). 1A23 (pMA5L) and 1A23 (pMA5S) were cultivated in the presence of xylose of different concentrations [0, 0.5, 1.0, 1.5, 2.0, 2.5, 3.0, 3.5, 4.0, 4.5 and 5.0% (w/v)]. The addition of xylose within a suitable concentration range greatly enhanced the α-amylase activity of both AmyL and AmyS, and the maximal α-amylase activity of AmyL and AmyS in the medium were obtained when 4.0% xylose was added (Figure 3). The result showed that α-amylase secretion was dependent very closely on PrsA expression level whether expressed at a high level (AmyL) or at a low level (AmyS).

### Combinatorial overexpression of PrsA combined with the screened components

**Figure 4:**
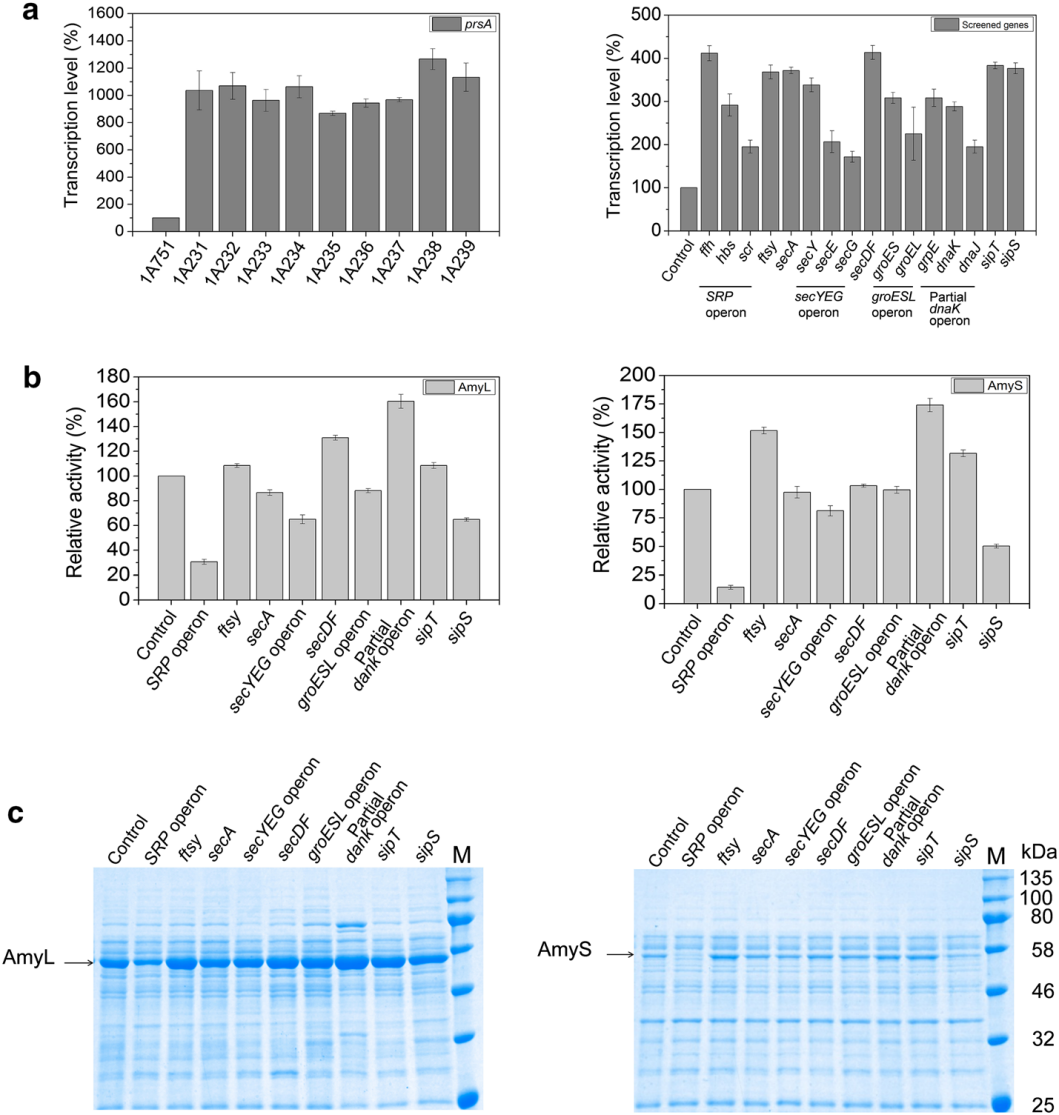
Overexpression of PrsA combined with the screened components in *B. subtilis*. **a** The transcriptional levels of the genes or gene operons in *B. subtilis*. The first figure shows the transcriptional level of *prsA* in recombinant strains; the transcription level of 1A751 (control) is regarded as 100%. The second figure indicates the transcriptional levels of nine genes or gene operons in nine corresponding strains, respectively; Control, the transcriptional levels of nine genes or gene operons (all regarded as 100%) in 1A751. **b** Analysis of α-amylase (AmyL and AmyS) activity in nine combinational overexpression strains. The α-amylase activity was converted to percent increase referring to the respective control strains [the former, 1A23 (pMA5L); the latter, 1A23 (pMA5S)]. **c** SDS-PAGE analysis of α-amylase (AmyL and AmyS) in nine combinational overexpression strains. *Lane M* molecular weight marker. The former *Control* is 1A23 (pMA5L); the latter *Control* is 1A23 (pMA5S). Specifically, the genes or gene operons *SRP*, *ftsy*, *secA*, *secYEG*, *secDF*, *groESL* operon, partial *dnaK* operon, *sipT* and *sipS* in **a**, **b** and **c** corresponded to strains 1A231, 1A232, 1A233, 1A234, 1A235, 1A236, 1A237, 1A238 and 1A239, respectively.

Following identification of PrsA as a main engineering target and the optimization of PrsA overexpression level, we next undertook the combinatorial overexpression of pairs of Sec pathway genes in order to unveil further bottlenecks of heterologous protein production and secretion. For this goal, nine genes or gene operons *SRP*, *ftsy*, *secA*, *secYEG*, *secDF*, *groESL* operon, partial *dnaK* operon, *sipT* and *sipS*, overexpression of which slightly enhanced the production and secretion of AmyL or AmyS, were screened to be overexpressed combined with PrsA, respectively. Therefore, we generated nine double-overexpression strains as follows: 1A231 (*prsA SRP*), 1A232 (*prsA ftsy*), 1A233 (*prsA secA*), 1A234 (*prsA secYEG*), 1A235 (*prsA secDF*), 1A236 (*prsA groESL* opeon), 1A237 (*prsA* partial *dnaK* operon), 1A238 (*prsA sipT*), 1A239 (*prsA sipS*), by transforming *Ahd*I-lineared corresponding integration vectors into 1A751. The nine genes or gene operons were all under the control of P_*grac*_, and the *prsA* gene was still under the control of P_*xylA*_. By real-time quantitative PCR, the transcriptional levels of *prsA* (with the addition of 4.0% xylose) and other nine screened genes in corresponding recombinant strains were much higher than that in control strain 1A751 (Figure 4a).

The expression plasmids pMA5L and pMA5S were transformed into the nine recombinant strains, respectively. The obtained strains were grown in liquid SR medium in the presence of 4.0% xylose, and the α-amylase activity of AmyL and AmyS in medium of corresponding recombinant strains were measured compared with the strain 1A23 (pMA5L or pMA5S) at 72 h (Figure 4b). For AmyL production, the overexpression of *prsA* combined with partial *dnaK* operon, *secDF*, *ftsy* and *sipT*, respectively, improved the activity of α-amylase in medium in different extent (160, 131, 108 and 108%, respectively; *t* test, all P < 0.05). As to AmyS production, the overexpression of *prsA* combined with partial *dnaK* operon, *ftsy* and *sipT* respectively, resulted in moderate increase (173, 151 and 132%, respectively; *t* test, all P < 0.05) of α-amylase in medium. It can be seen that when *prsA* was overexpressed combined with partial *dnaK* operon, the α-amylase activity of AmyL and AmyS in medium both were the highest, indicating that the chaperones of Dnak series play a vital role in α-amylase production.

To provide further evidence supporting the results described above, the SDS-PAGE analysis was performed to compare the production of α-amylase. Distinct bands with a molecular mass of about 55 kDa were observed which was in good agreement with the deduced value in both AmyL and AmyS production strains, which was consistent with the results as previously mentioned (Figure 4c).

### Characterization of engineered vs. parental strains on α-amylase production

**Figure 5:**
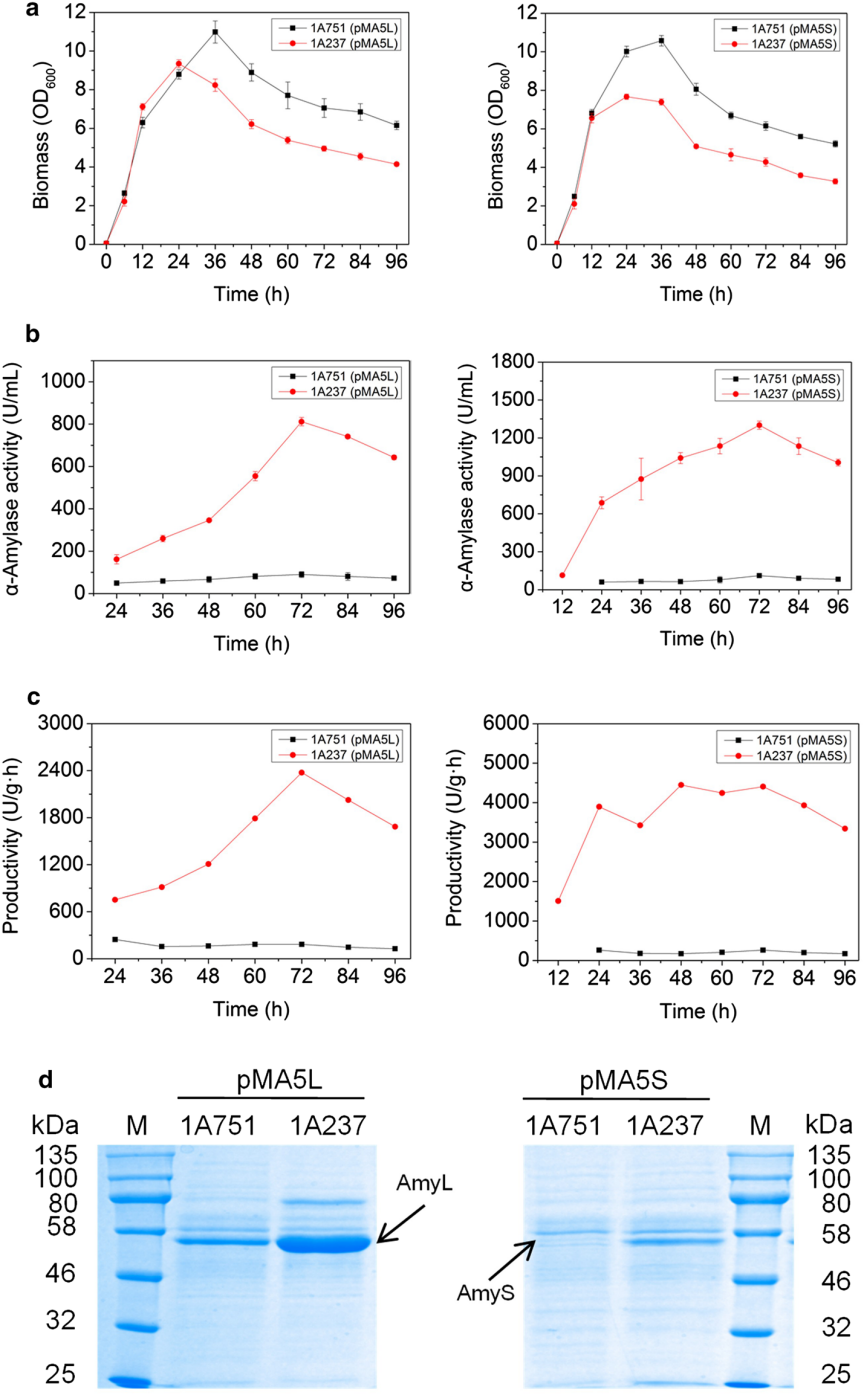
Characterization of engineered and parental strains. *B. subtilis* 1A751 (pMA5L) (parental strain), 1A237 (pMA5L) (engineered strain), 1A751 (pMA5S) (parental strain) and 1A237 (pMA5S) (engineered strain) were cultivated for 96 h at 37°C in 30 mL SR medium supplemented with 4% xylose (w/v). **a** Growth of the strains. **b** Analysis of α-amylase (AmyL and AmyS) activity. **c** Analysis of productivity. **d** SDS-PAGE analysis of α-amylase (AmyL and AmyS) at 72 h.

We chose recombinant strain 1A237 overexpressing *prsA* and partial *dnaK* operon for detailed analyses on growth condition and α-amylase (AmyL and AmyS) production, and, meanwhile, compared them with the parental strain 1A751. Whether producing AmyL or AmyS, 1A237 and 1A751 have similar biomass during the early stage of exponential growth (<12 h); however, they differed significantly upon entry into the late stage of exponential growth (Figure 5a). The OD_600_ values of 1A237 peaked at 24 h and were lower than that of 1A751, which continued to increase after 12 h and peaked at 36 h. Nevertheless, simultaneous overexpression of *prsA* and *dnaK* operon improved the final α-amylase activity of AmyL and AmyS by about ninefold and 12-fold (Figure 5b). The results of SDS-PAGE analysis of α-amylase in medium were consistent with the activity data (Figure 5d). Furthermore, *B. subtilis* 1A237 had much higher productivity of AmyL and AmyS (approximately 13- and 17-fold, respectively) than that of the parental strain 1A751 (Figure 5c).

As shown above, AmyS producer exhibited generally greater increases of yield compared to AmyL producer, indicating that this, the protein of initially lower production, has more room for phenotype improvement. In addition, phenotypic characterization of engineered α-amylase producers revealed that higher α-amylase production was accompanied by reduced growth rates. Specifically, growth rates were determined as μ = 0.30 h^−1^ for 1A751 (pMA5-AmyL), μ = 0.23 h^−1^ for 1A237 (pMA5-AmyL), μ = 0.29 h^−1^ for 1A751 (pMA5-AmyS), and μ = 0.21 h^−1^ for 1A237 (pMA5-AmyS). The negative impact of α-amylase production on the growth rate was also observed in various other engineered strains of this study (data not shown).

### Production of α-amylase (AmyL and AmyS) in 7.5 L fermentor with fed-batch strategy

**Figure 6:**
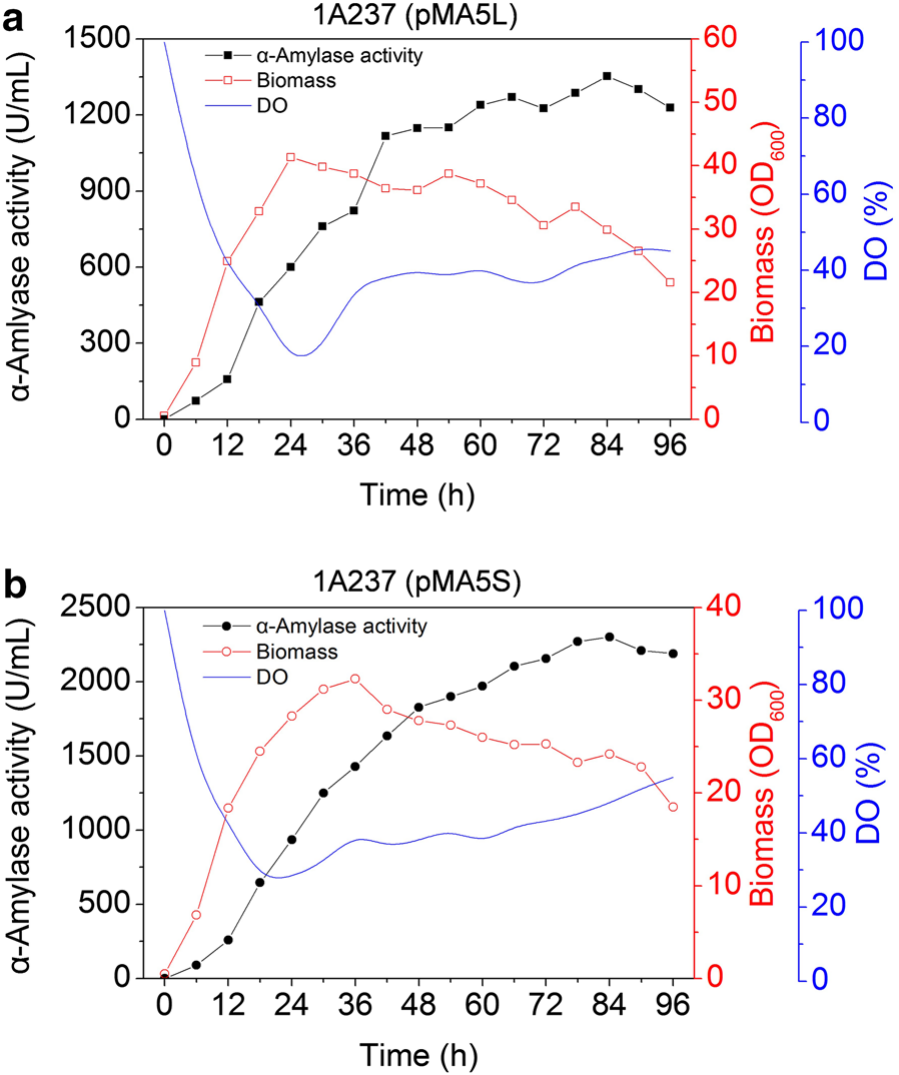
Production of α-amylase in recombinant strain 1A237 by fed-batch fermentation in 7.5 L fermentor. **a** AmyL production. *Solid square* α-amylase activity in medium. *Hollow square* biomass. *Blue line* DO concentration. **b** AmyS production. *Solid circle* α-amylase activity in medium. *Hollow circle* biomass. *Blue line* DO concentration.

The expression efficiency of *B. subtilis* 1A237 (pMA5-AmyL) and 1A237 (pMA5-AmyS) were further explored in 7.5 L fermentor, respectively. The fermentor was inoculated with 5% (v/v) of freshly cultured 1A237 (pMA5-AmyL) or 1A237 (pMA5-AmyS) grown in SR medium at 37°C for 18 h. To maintain cell growth and α-amylase production, we choose a fed-batch strategy. When the cell growth rate was constant, 8.0% (w/v) soluble starch was added at a constant flow rate until the final concentration of soluble starch was up to 4.0% (w/v). For AmyL production (Figure 6a), during the growth phase, the maximum biomass in the fermentor reached 41.3 (OD_600_) at 24 h. The activity of α-amylase in medium was continuously increased and reached the maximum of 1,352 U/mL at 84 h with a high productivity of 16.1 U/mL h. For AmyS production (Figure 6b), the maximum biomass in the fermentor reached 32.3 (OD_600_) at 36 h. The activity of α-amylase in medium was continuously increased and reached the maximum of 2,300 U/mL at 84 h with a high productivity of 27.4 U/mL h. The high activity of α-amylase indicated that *B. subtilis* was a suitable host for the industrial production of α-amylase.

## Discussion

In the present work, to produce two heterologous proteins (AmyL and AmyS) in *B. subtilis*, *amyl* and *amys* were equipped with their native signal peptide SP_*amyl*_ and SP_*amyS*_, respectively, and under the control of the promoter P_*HpaII*_. SP_*amyl*_ and SP_*amyS*_ are Sec-dependent signal peptides, so that AmyL and AmyS can traverse the cytoplasmic membrane via the Sec-translocon. P_*HpaII*_ is a widely used, strong and constitutive promoter from *Staphylococcus aureus*. The recombinant AmyL was secreted into the culture medium by *B. subtilis* 1A751 in a markedly higher expression level compared with AmyS (Figure 1c). Whereas, there was no AmyL or AmyS accumulation in the cell fraction, which indicates that nearly the whole AmyL or AmyS can be directed into the medium and SP_*amyl*_ and SP_*amyS*_ are efficient signal peptides for AmyL and AmyS secretion, respectively. However, the α-amylase activity of AmyL or AmyS in 1A751 was still low, compared to some public studies [32, 33]. Researches show that high gene expression level may result in saturation of the Sec-translocon capacity; that is to say, the expression level of the components involved in Sec pathway may be insufficient for secretion of heterologous proteins. To improve the yields of heterologous proteins, it is necessary to identify the bottlenecks hampering their production.

Sec pathway is the major route for protein secretion in *B. subtilis*. The components involved in Sec pathway are roughly divided into four categories: Signal recognition particle, translocase complex, signal peptidases and chaperones. We evaluated the single overexpression of 23 genes or gene operons involved in Sec pathway on the production of AmyL and AmyS, respectively, and found that *prsA* overexpression markedly improved the production of both AmyL (3.2-fold) and AmyS (5.5-fold) (Figure 2). The results point to PrsA as a bottleneck for the secretion of AmyL and AmyS, which is consistent with previous studies [31, 34]. PrsA is an extracellular folding chaperone that influences protein folding in the (pseudo) periplasmic space. In this environment the lipoprotein PrsA is required for protein stability in the post-translocational phase of secretion [31, 34, 35]. AmyL and AmyS are heterologous proteins from *B. licheniformis* and *B. stearothermophilus*, respectively. With the overexpression of *prsA*, more α-amylase (AmyL or AmyS) was rapidly folded into its native conformation, allowing α-amylase to gain resistance against extracellular proteases. In addition, the result that AmyL expressed at relative high level and AmyS expressed at relative low level were both significantly enhanced by overexpression of *prsA* was consistent with previous studies [31]. On account of the key role of PrsA in the process of protein secretion, we modulated the expression levels of *prsA*. With the increase of xylose concentration, the expression level of *prsA* was improved. The α-amylase activity of AmyL and AmyS were highest when the concentration of xylose was 4.0%. This indicates that there is an optimal expression level of *prsA* for heterologous protein secretion in *B. subtilis*, as stated in the research that there was a linear correlation between the number of cellular PrsA molecules and the number of secreted AmyQ molecules over a wide range of *prsA* and *amyQ* expression levels [31]. In addition, the single overexpression of *SRP*, *ftsy*, *secA*, *secYEG*, *secDF*, *groESL* operon, partial *dnaK* operon, *sipT* and *sipS* moderately or marginally improved the production of AmyL or AmyS. However, the contribution of genes to AmyL and AmyS secretion were not completely consistent. For example, *secYEG* overexpression improved the production of AmyL but not AmyS; *groESL* operon and *sipT* overexpression enhanced the secretion of AmyS, but reduced AmyL production. From these results, we can know that partial components of Sec pathway have different impacts on different proteins secretion.

Based on the optimal expression level of *prsA*, we overexpressed 9 screened genes or gene operons combined with *prsA* in 1A751, respectively. The overexpression of *prsA* and partial *dnaK* operon resulted in the highest α-amylase activity of both AmyL and AmyS. Dnak series are intracellular molecular chaperones mediating protein folding, minimizing aggregation and maintaining pre-proteins in translocation-competent conformations. With the overexpression of partial *dnaK* operon, the pre-proteins obtained stronger resistance against intracellular proteases, resulting in further improvement of heterologous protein secretion. Unexpectedly, the influence of the double-overexpression strains was not always consistent with the performance of the respective single-overexpression strain. For example, we predicted that overexpression of *prsA* and *secDF* would contribute the most to AmyL production compared with other gene combinations; however, the α-amylase activity of 1A237 (*prsA* partial *dnaK* operon) was the highest and exceeded that of 1A235 (*prsA secDF*) by 22%. In addition, the α-amylase activity of AmyL in medium of 1A231 (*prsA SRP*), 1A233 (*prsA secA*), 1A234 (*prsA secYEG*), 1A236 (*prsA groESL* opeon) and 1A239 (*prsA sipS*) were even markedly lower than that of 1A23 (*prsA*). The similar phenomenon also appeared when AmyS was produced. Although the α-amylase activity of AmyS in medium of 1A237 (*prsA* partial *dnaK* operon) was the highest as predicted, the α-amylase activity of AmyS in medium of 1A235 (*prsAsecDF*) was almost the same with that of 1A23 (*prsA*). Double-overexpression of *prsA*-*secA*, *prsA*-*groESL* operon, *prsA*-*ftsy*, *prsA*-*SRP* and *prsA*-*sipS* even reduced the production of AmyS compared with single overexpression of *prsA*. Particularly, with the overexpression of *SRP* operon, the secretion of AmyL and AmyS was severely impaired, which is unexpected. We suspect that excessive SRP may cause some unknown harmful effect on physiological characteristic of *B. subtilis*, resulting into remarkably decrease of α-amylase production. In short, the overexpression of *prsA* combined with some genes or gene operons which have positive effect on α-amylase secretion reduced the production of AmyL or AmyS in some extent. These observations suggest that the interactions between components of Sec pathway have different effects on protein secretion.

By comparing the characterization of engineered and parental strains, we can see simultaneous overexpression of *prsA* and partial *dnaK* operon improved the final α-amylase activity of AmyL and AmyS by about ninefold and 12-fold and the productivity of AmyL and AmyS by approximately 13- and 17-fold, respectively. For heterologous protein secretion, a key observation is that proteins must fold rapidly if they are to avoid blocking the translocase itself or cell-wall growth sites: slowly folding proteins expose protease-sensitive sites that are not exposed in the fully folded protein. This is best illustrated by the kinetics of secretion of AmyL [36]. Pulse-chase experiments show that during secretion from *B. licheniformis* almost 100% of the synthesized protein is recovered from the culture medium. By contrast, when transferred to *B. subtilis*, ~75% of the initially formed protein is degraded, and only 25% is recovered from the growth medium. The intracellular molecular chaperones (mainly DnaK series) may potentially assist AmyL and amyS to adopt loosely folded conformations that are compatible with the secretion apparatus and/or to attain configurations that are less susceptible to intracellular protease degradation. After membrane translocation, the secreted AmyL or AmyS is presumably refolded. The rate of protein refolding has been shown to affect the final production yield of a secretory protein [37]. Usually, a higher production yield can be attained for secretory proteins with a higher refolding rate. Increasing the production of PrsA can quicken the rate of protein refolding and improve the recovery of limited number of proteins that are substrates for this chaperone, presumably by reducing their susceptibility to extracellular proteolysis. In a word, With the combinational overexpression of both intracellular and extracytoplasmic molecular chaperones, the pre-protein and mature protein both have stronger resistance against proteases, resulting in high yields of heterologous protein in *B. subtilis*.

## Conclusion

We have shown that the combinational overexpression of *prsA* and partial *dnaK* operon significantly improved the production of AmyL and AmyS, suggesting that the stability of protein is vital for protein secretion and the deficiency of intracellular and extracellular chaperones may be main bottleneck of heterologous protein production in *B. subtilis*. It can thus be seen that balanced expression of Sec pathway components is crucial for efficient translocation and secretion. Moreover, we need to find out in detail how components of Sec pathway interact with each other, so that the interactions can be optimized specifically to improve the secretion of heterologous protein in *B. subtilis*.

## Methods

### Bacterial strains, plasmids and growth conditions

Bacterial strains and plasmids used in this study are listed in Tables 1 and 2, respectively. The bacterial strain *Bacillus licheniformis* CICC 10181 and *Geobacillus stearothermophilus* ATCC 31195 were used as the source of the AmyL (*amyl*) gene and AmyS (*amys*) gene, respectively. *Escherichia coli* DH5α served as a host for cloning and plasmid preparation. *Bacillus subtilis* 1A751, which is deficient in two extracellular proteases (*nprE, aprE*), was used as a host for expression of AmyL and AmyS. The plasmid pMA5 is an *E. coli/B. subtilis* shuttle vector and used to clone and express AmyL and AmyS. The plasmid pDD is an integration vector which is derivative from pDL and just contains the front and back parts of *amyE*. Transformants of *E. coli* and *B. subtilis* were selected on Luria–Bertani (LB) agar [1% (w/v) Trytone, 0.5% (w/v) Yeast extract, 1% (w/v) NaCl and 2% (w/v) agar], supplemented with ampicillin (100 μg/mL) or kanamycin (50 μg/mL) depending on the plasmid antibiotic marker. Unless otherwise specified, integrated *B. subtilis* mutants were selected on LB agar, containing kanamycin (50 μg/mL) or chloramphenicol (12.5 μg/mL). *E. coli* DH5α was incubated in LB medium supplemented with ampicillin (100 μg/mL) at 37°C. *B. subtilis* was cultivated in SR medium [1.5% (w/v) tryptone, 2.5% (w/v) yeast extract and 0.3% (w/v) K_2_HPO_4_, pH 7.2] contained additionally kanamycin (50 μg/mL) or chloramphenicol (12.5 μg/mL) at 37°C. All of the strains were incubated under a shaking condition at 200 rpm. All of the experiments were repeated at least three times and mean values were used for comparison.

### Primers and oligonucleotides

Polymerase chain reaction (PCR) primers and oligonucleotides used in this study were synthesized by GENEWIZ (Suzhou, China) and listed in Additional file 2: Table S1.

### General manipulation

Standard molecular techniques including *E. coli* transformation were carried out according to Sambrook et al. [38]. *B. subtilis* was naturally transformed using “Paris Method” [39, 40]. PCRs were performed using PrimeSTAR Max DNA Polymerase (TaKaRa, Japan). DNA fragments and PCR products were excised from a 0.8% agarose gel and purified by E.Z.N.A.™ Gel Extraction Kit (200) (Omega Bio-tek, Inc., USA) according to the manufactures’ instruction. E.Z.N.A.™ Plasmid Mini Kit I (Omega Bio-tek, Inc., USA) was applied for plasmid extraction according to the manufactures’ instruction. Genomic DNA isolation was carried out by TIANamp Bacteria DNA Kit (TIANGEN BIOTECH (BEIJING) CO., LTD., China). All the DNA constructs were sequenced by GENEWIZ (Suzhou, China).

### Construction and transformation of the plasmid for secreted AmyL or AmyS

Based on the nucleotide sequence of the gene encoding AmyL or AmyS, the primer pairs amyL-F/amyL-R or amyS-F/amyS-R (Additional file 2: Table S1) were designed to amplify the gene *amyl* or *amys* from *Bacillus licheniformis* CICC 10181 or *Geobacillus stearothermophilus* ATCC 31195 with the introduction of *Eco*RI and *Xho*I restriction site at 5′ of the forward and reverse primers, respectively. The gene *amyl* or *amys* contains their native signal peptide SP_*amyl*_ or SP_*amys*_. The *Eco*RI-*Xho*I digested fragments *amyl* and *amys* were ligated with pET28-a(+) linearized by the same enzymes, resulting in pET28L and pET28S under the control of the T7-promoter and T7-terminator, respectively.

The DNA fragments AmyL gene-T7 terminator (*amyl*-T7) and AmyS gene-T7 terminator (*amys*-T7) were amplified from pET28L and pET28S plasmids as template with the primer pairs amyLT-F/R and amyST-F/R, which contained *Nde*I and *Bam*HI restriction site at 5′ of the forward and reverse primers, respectively (Additional file 1: Figure S1). It was followed by ligation of the *Nde*I-*Bam*HI digested *amyl*-T7 or *amys*-T7 fragment with pMA5 linearized by the same enzymes, which is an *E. coli/B. subtilis* shuttle vector containing an widely used strong promoter P_*HpaII*_ and a strong *B. subtilis* ribosome binding site (RBS), resulting in the recombinant plasmid pMA5L or pMA5S. *B. subtilis* 1A751 were transformed according to the method as previously described with the control plasmid pMA5 and expression plasmids pMA5L and pMA5S, respectively.

### Construction of the integration vectors for genes overexpression

All the integration vectors (Table 2) used in this study were constructed by a sequence-independent “simple cloning” method without the need for restriction and ligation enzymes [41]. To develop an integration-vector system for co-overexpression of two different genes, we constructed an integration plasmid pDDXG in advance. First of all, a 1.4-kb insertion fragment (the promoter P_*xylA*_) was subcloned into an 8.1-kb pDD vector backbone, yielding a 9.5-kb plasmid, pDDX. The linear vector backbone was amplified by using the forward primer pDD-F (5′GTTCACTTAAA TCAAAGGGGGAAAT**AGAAGTCTCGTTCCGACAGTTGGCA**3′) and the reverse primer pDD-R (5′**CGTTTTACAACGTCGTGACTGGGAAA**CCGGGAAT TCTCAT GTTTGACAGC3′). pDD-F/R contain the last 25 bp of the 3′ terminus of the insertion sequence (underlined) and the first 25 bp of the 5′ terminus of the vector sequence (bold). Similarly, the insertion fragment was amplified by primers xylA-F (5′**AAG CTGTCAAACATGAGAATTCCCGG**TTTCCCAGTCACGACGT TGTAAAAC3′) and xylA-R (5′TGCCAACTGTCGGAACGAGACTTCT**ATTT CCCCCTTTGATTTAAGTGAAC**3′). xylA-F/R have the reverse complementary sequences of pDD-F/R, respectively. Then, the DNA multimer is generated based on these DNA templates by prolonged overlap extension PCR (POE-PCR). Eventually, the POE-PCR products (DNA multimer) were transformed into competent *E. coli* DH5α directly, yielding the recombinant plasmid pDDX. Using primers grac-F/R and pDDX-F/R, the P_*grac*_ fragment and linear vector backbone were amplified from pHT43 and pDDX, respectively. Subsequently, the integration vector pDDXG was constructed as described above. In the same way, pDDXBG and pDDXGB into which the gene bgaB was inserted under the control of P_*xylA*_ and P_*grac*_, respectively, were also constructed.

All the integration vectors for overexpression of the corresponding single gene or gene operon involved in Sec pathway in *B. subtilis* were constructed based on the plasmid pDDXG. 19 genes (*ffh, hbs, scr, ftsy, csaA, secA, secY, secE, secG, secDF, yrbF, spoIIIJ, yqjG, sipT, sipS, sipU, sipV, sipW and prsA*) and two gene operons (*groESL* operon and partial *dnaK* operon) were amplified using corresponding primers (Additional file 2: Table S1) and *B. subtilis* 168 genomic DNA as the template. Meanwhile, two artificial gene operons (*SRP* operon and *secYEG* operon) were assembled. The genes *ffh*, *hbs* (containing its own ribosome-binding site) and *scr* were amplified using primers ffh-F2/R2, hbs-F2/R2 and scr-F2/R2, respectively, and then the *SRP* operon was assembled by POE-PCR using primers ffh-F2/scr-R2. The genes *secY*, *secE* (containing its own ribosome-binding site) and *secG* (containing its own ribosome-binding site) were amplified using primers secY-F2/R2, secE-F2/R2 and secG-F2/R2, respectively, and the *secYEG* operon was assembled by POE-PCR using primers secY-F2/secG-R2. The DNA fragments of 19 genes and 4 gene operons above were then inserted into the plasmid pDDXG, successively, under the control of the promoter P_*xylA*_, resulting into 23 corresponding integration vectors (Table 2).

The five genes *ftsy*, *secA*, *secDF*, *sipT* and *sipS* and four operons *SRP* operon, *secYEG* operon, *groESL* operon and partial *dnaK* operon were amplified by PCR from genomic DNA of *B. subtilis* 168 or corresponding plasmids using primers (Additional file 2: Table S1). The vector backbone was amplified from pDD23 with primers pDD23-F/R. By the “simple cloning” method as described above, nine corresponding integration plasmids were constructed successfully.

### Isolation of total RNA and Real-time PCR

The culture was harvested at 48 h. Total RNA of bacteria was isolated by using SV total RNA isolation kit (Cat. Z3100, Promega). The cDNA chain was synthesized by using Reverse Transcription System (Cat. A3500, Promega). Real-time PCR was performed by using Real time PCR Kit (Cat. DRR041 S, TaKaRa). The target genes were amplified using respective primers. *B. subtilis* 16 s rDNA was amplified as control and the PCR protocol was as follows: 2 min at 50°C, 10 min at 95°C, and then 35 cycles consisting of 45 s at 95°C, 1 min at 52°C, and 30 s at 72°C. Reactions were carried out in real-time PCR detection system (IQ5, Bio-RAD).

### Fed-batch fermentation in 7.5 L fermentor

The recombinant *B. subtilis* 1A751 (pMA5L) and 1A751 (pMA5S) were used to scale up fermentation in 7.5 L BIO FLO 310 fermentor (New Brunswick Scientific co Inc., USA), with a fed-batch strategy. The airflow rate was 6.0 L/min, and dissolved oxygen tension was maintained between 20 and 40% air saturation by automatic adjustment of speed of the stirrer. The temperature was kept at 37°C and the pH was controlled at pH 7.0. Foam was controlled by the addition of a silicone-based anti-foaming agent. The fermentation medium (1.5% tryptone, 2.5% yeast extract, 2.0% soluble starch and 0.3% K_2_HPO_4_) was supplemented with 50 μg kanamycin/mL and 100 μg chloramphenicol/mL. The fermentation was performed with an initial working volume of 3.5 L. When the cell growth rate was constant, the substrate fed-batch mode was started by adding 8.0% soluble starch at a constant flow rate, until the final concentration of soluble starch was up to 4.0%. Cell growth was monitored by measuring OD_600_ of the fermentation broth. The activity of α-amylase was determined by measuring the supernatant of broth.

### Enzyme assays

The definition of α-amylase is described below. One unit of enzyme is the amount of amylase needed to complete the hydrolysis of starch into dextrin per minute at 70°C and pH 6.0. The measurement was done according to Chinese Industrial Standard (GB 8275-2009). The calculation of the enzyme activity was based on the formula below. *X* = *c* × *n* × 16.67, where *X* is the enzyme activity of the sample (U/mL), *c* is the concentration of the control enzyme (U/mL) corresponded with the absorbance and *n* is the dilution fold. β-galactosidase assay was measured as described [42].

### SDS-PAGE analysis

Culture samples (1 mL) were harvested and the supernatant was separated from the culture medium by centrifugation (12,000*g*, 10 min, 4°C). After adding 5× SDS-PAGE sample buffer, the supernatants were boiled for 10 min and proteins were separated in SDS-PAGE using the NuPAGE 10% Bis–Tris Gel (Novex by Life Technologies, USA) in combination with MOPS SDS Running Buffer (Invitrogen Life Technologies, USA). PageRuler Prestained Protein Ladder (Invitrogen Life Technologies, USA) was used to determine the apparent molecular weight of separated proteins. Proteins were visualized with Coomassie Brilliant Blue.

## Additional files

Additional file 1:
**Figure S1.** The construction of all the integration plasmids used in this study. Additional file 2:
**Table S1.** Primers used in this study.
