# Supplementary figures and images for: Combinatorial Sec pathway analysis for improved heterologous protein secretion in Bacillus subtilis: identification of bottlenecks by systematic gene overexpression
Source: Microb Cell Fact. 2015 Jun 26;14:92. doi: 10.1186/s12934-015-0282-9 (PMC4482152; doi:10.1186/s12934-015-0282-9)

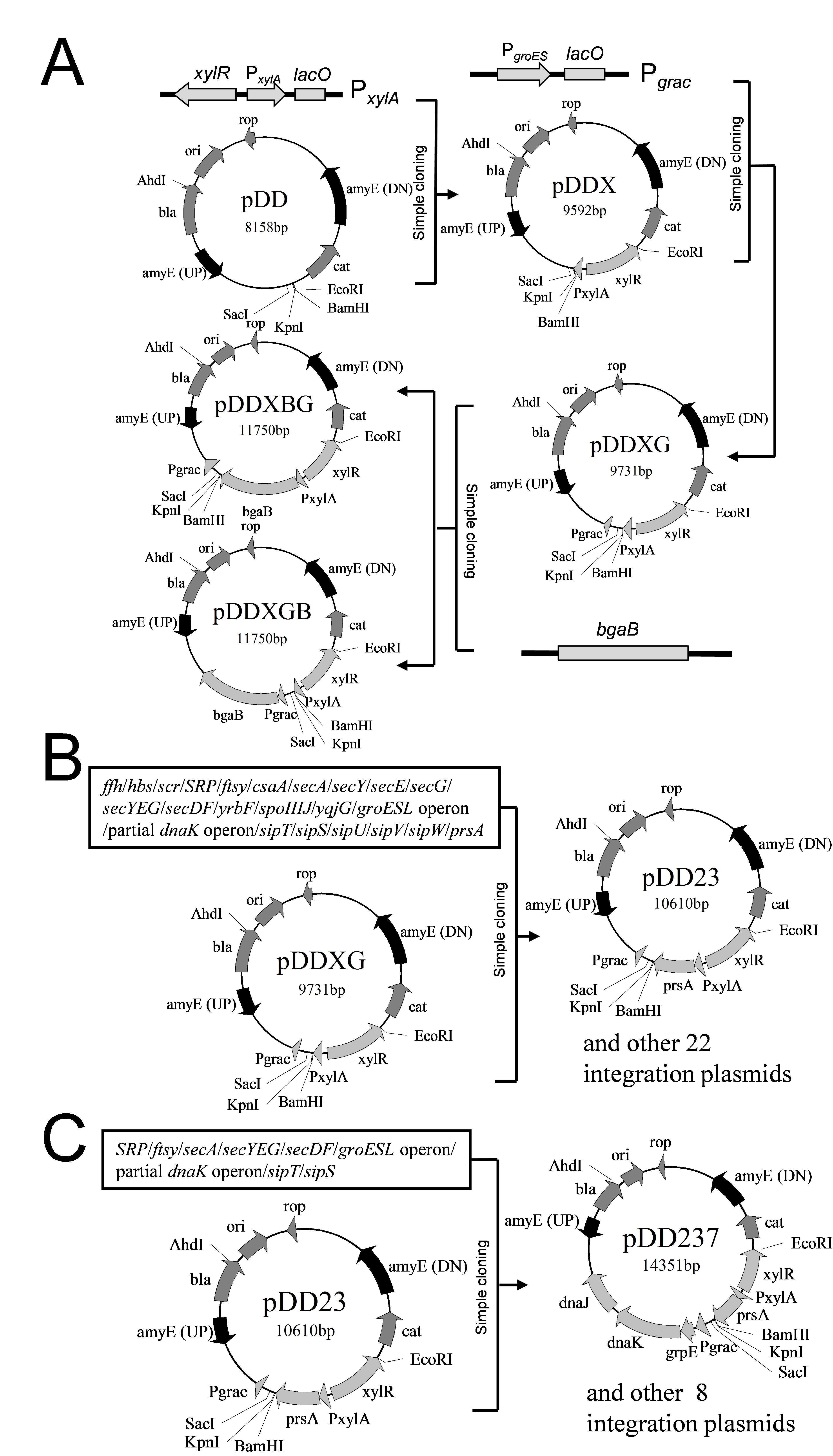

Supplement: Additional file 1: — Figure S1. The construction of all the integration plasmids used in this study. [file 12934_2015_282_MOESM1_ESM.jpg]
