# Supplementary material for: Combinatorial Sec pathway analysis for improved heterologous protein secretion in Bacillus subtilis: identification of bottlenecks by systematic gene overexpression
Source: Microb Cell Fact. 2015 Jun 26;14:92. doi: 10.1186/s12934-015-0282-9 (PMC4482152; doi:10.1186/s12934-015-0282-9)
Supplement: Additional file 2: — Table S1. Primers used in this study. [file 12934_2015_282_MOESM2_ESM.docx]

**Additional file: Table S1**

Primers used in this study

| Prmier | Sequence(5’-3’) |
| --- | --- |
| amyL-F | GAGCTCATGAAACAACAAAAACGGCTTTATG |
| amyL-R | CTCGAGCTATCTTTGAACATAGATCGAAACC |
| amyS-F | GAGCTCATGGATATTGAAAACGATTACAAA |
| amyS-R | CTCGAGAGGTGAATTTCGACCTCTAGAACG |
| amyLT-F | CATATGATGAAACAACAAAAACGGCTTTATG |
| amyLT-R | GGATCCCAAAAAACCCCTCAAGACCCGTTTAG |
| amyST-F | CATATGATGGATATTGAAAACGATTACAAA |
| amyST-R | GGATCCCAAAAAACCCCTCAAGACCCGTTTAG |
| xylA-F | AAGCTGTCAAACATGAGAATTCCCGGTTTCCCAGTCACGACGTTGTAAAAC |
| xylA-R | TGCCAACTGTCGGAACGAGACTTCTATTTCCCCCTTTGATTTAAGTGAAC |
| pDD-F | GTTCACTTAAATCAAAGGGGGAAATAGAAGTCTCGTTCCGACAGTTGGCA |
| pDD-R | CGTTTTACAACGTCGTGACTGGGAAACCGGGAATTCTCATGTTTGACAGC |
| grac-F | GCATCGAGCTGGGTAATAAGCGTTGAAAGGAGGTAAGGATCACTAG |
| grac-R | AAAGCCTGACTGGCGGTTAAATTGCTTCCTCCTTTAATTGGTGTTGGTTG |
| pDDX-F | CAACCAACACCAATTAAAGGAGGAAGCAATTTAACCGCCAGTCAGGCTTT |
| pDDX-R | CTAGTGATCCTTACCTCCTTTCAACGCTTATTACCCAGCTCGATGC |
| bgaB-F1 | GTTCACTTAAATCAAAGGGGGAAATATGAATGTGTTATCCTCAATTTG |
| bgaB-R1 | TGCCAACTGTCGGAACGAGACTTCTCTAAACCTTCCCGGCTTCATCATGC |
| pDDXG-F1 | GCATGATGAAGCCGGGAAGGTTTAGAGAAGTCTCGTTCCGACAGTTGGCA |
| pDDXG-R1 | CAAATTGAGGATAACACATTCATATTTCCCCCTTTGATTTAAGTGAAC |
| bgaB-F2 | CAACCAACACCAATTAAAGGAGGAAATGAATGTGTTATCCTCAATTTG |
| bgaB-R2 | AAAGCCTGACTGGCGGTTAAATTGCCTAAACCTTCCCGGCTTCATCATGC |
| pDDXG-F2 | GCATGATGAAGCCGGGAAGGTTTAGGCAATTTAACCGCCAGTCAGGCTTTC |
| pDDXG-R2 | CAAATTGAGGATAACACATTCATTTCCTCCTTTAATTGGTGTTGGTTG |
| ffh-F | GTTCACTTAAATCAAAGGGGGAAATATGGCATTTGAAGGATTAGCCGACC |
| ffh-R | TGCCAACTGTCGGAACGAGACTTCTTTACATAAAAGGTAGCTTAAACCCT |
| hbs-F | GTTCACTTAAATCAAAGGGGGAAATATGAACAAAACAGAACTTATCAATG |
| hbs-R | TGCCAACTGTCGGAACGAGACTTCTTTATTTTCCGGCAACTGCGTCTTTA |
| scr-F | GTTCACTTAAATCAAAGGGGGAAATGCATCGTAATAGATGCAACATAAAATTTG |
| scr-R | TGCCAACTGTCGGAACGAGACTTCTCCCATTATACATGAACCTTGTTTCG |
| ffh-F2 | GTTCACTTAAATCAAAGGGGGAAATATGGCATTTGAAGGATTAGCCGACC |
| ffh-R2 | CATTGATAAGTTCTGTTTTGTTCAT TTACATAAAAGGTAGCTTAAACCCT |
| hbs-F2 | GGTTTAAGCTACCTTTTATGTAATTTGGGAGGAGGTGAAAGGCATG |
| hbs-R2 | CAAATTTTATGTTGCATCTATTACGATGCTTATTTTCCGGCAACTGCGTC |
| scr-F2 | TAAAGACGCAGTTGCCGGAAAATAAGCATCGTAATAGATGCAACATAAAATTTG |
| scr-R2 | TGCCAACTGTCGGAACGAGACTTCTCCCATTATACATGAACCTTGTTTCG |
| ftsy-F | GTTCACTTAAATCAAAGGGGGAAATATGAGCTTTTTTAAAAAATTAAAAG |
| ftsy-R | TGCCAACTGTCGGAACGAGACTTCTTTAATCGTCGGCTTTTTCCACTAAATC |
| csaA-F | GTTCACTTAAATCAAAGGGGGAAATATGGCAGTTATTGATGACTTTGAG |
| csaA-R | TGCCAACTGTCGGAACGAGACTTCTTTATCCGATTTTTGTGCCGTTTGG |
| secA-F | GTTCACTTAAATCAAAGGGGGAAATATGCTTGGAATTTTAAATAAAATG |
| secA-R | TGCCAACTGTCGGAACGAGACTTCTCTATTCAGTACGGCCGCAGC |
| secY-F | GTTCACTTAAATCAAAGGGGGAAATTTGTTTAAAACAATCTCCAAC |
| secY-R | TGCCAACTGTCGGAACGAGACTTCTCTAGTTTTTCATAAATCCACGGTAG |
| secE-F | GTTCACTTAAATCAAAGGGGGAAATATGCGTATTATGAAATTCTTTAAAG |
| secE-R | TGCCAACTGTCGGAACGAGACTTCTTTATTCAACTATTAAACGAATTAATTG |
| secG-F | GTTCACTTAAATCAAAGGGGGAAATATGCACGCAGTTTTGATTACCTTATTG |
| secG-R | TGCCAACTGTCGGAACGAGACTTCTCTATAGGATATAAGCAAGCGCAATCG |
| secY-F2 | GTTCACTTAAATCAAAGGGGGAAATTTGTTTAAAACAATCTCCAAC |
| secY-R2 | CGCATGTAAAAGACCTCCACAATTTCTAGTTTTTCATAAATCCACGGTAGTTTCG |
| secE-F2 | CTAGTTTTTCATAAATCCACGGTAGTAAATTGTGGAGGTCTTTTACATGC |
| secE-R2 | AATAAGGTAATCAAAACTGCGTGCATTTATTCAACTATTAAACGAATTAATTGAGAAATTCC |
| secG-F2 | CTCAATTAATTCGTTTAATAGTTGAATAATGAGTCTGGAGGTGTATGGGATG |
| secG-R2 | TGCCAACTGTCGGAACGAGACTTCTCTATAGGATATAAGCAAGCGCAATCG |
| secDF-F | GTTCACTTAAATCAAAGGGGGAAATATGAAAAAAGGACGCTTGATTGC |
| secDF-R | TGCCAACTGTCGGAACGAGACTTCTTTATTGCGCCGAATCTTTTTTCAG |
| yrbF-F | GTTCACTTAAATCAAAGGGGGAAATATGATGACTGGCACTTTAGGTAC |
| yrbF-R | TGCCAACTGTCGGAACGAGACTTCTTTATTCAGCAGCAGAAACTTCTC |
| spoIIIJ-F | GTTCACTTAAATCAAAGGGGGAAATATGTTGTTGAAAAGGAGAATAGG |
| spoIIIJ-R | TGCCAACTGTCGGAACGAGACTTCTTCACTTTTTCTTTCCTCCGGCAAA |
| yqjG-F | GTTCACTTAAATCAAAGGGGGAAATTTGTTAAAAACATATCAAAAACTTTTG |
| yqjG-R | TGCCAACTGTCGGAACGAGACTTCTTTATTTCACCGACTCAGTAAGAG |
| sipT-F | GTTCACTTAAATCAAAGGGGGAAATTTGACCGAGGAAAAAAATACGAATAC |
| sipT-R | TGCCAACTGTCGGAACGAGACTTCTTTATTTTGTTTGACGCATTTCG |
| sipS-F | GTTCACTTAAATCAAAGGGGGAAATTTGAAATCAGAAAATGTTTCGAAG |
| sipS-R | TGCCAACTGTCGGAACGAGACTTCTCTAATTTGTTTTGCGCATTTCG |
| sipU-F | GTTCACTTAAATCAAAGGGGGAAATTTGAATGCAAAAACAATCACG |
| sipU-R | TGCCAACTGTCGGAACGAGACTTCTTTATTTTGCCTGTCTCATCTCACC |
| sipV-F | GTTCACTTAAATCAAAGGGGGAAATATGAAAAAACGGTTTTGGTTTCTTG |
| sipV-R | TGCCAACTGTCGGAACGAGACTTCTTTATTCGGCATCAGAAATCACACC |
| sipW-F | GTTCACTTAAATCAAAGGGGGAAATATGAAGCTGATCAGTAATATTTTATACG |
| sipW-R | TGCCAACTGTCGGAACGAGACTTCTTTAAGTAGACATGGTGCTGTCCTTTG |
| groESL-F | GTTCACTTAAATCAAAGGGGGAAATTTGTTAAAGCCATTAGGTGATCG |
| groESL-R | TGCCAACTGTCGGAACGAGACTTCTTTACATCATTCCACCCATACC |
| dnak-F | GTTCACTTAAATCAAAGGGGGAAATATGTCAGAAGAAAAACAAACC |
| dnak-R | TGCCAACTGTCGGAACGAGACTTCTGGGTAATCATTGGGGTCGAGC |
| prsA-F | GTTCACTTAAATCAAAGGGGGAAATATGAAGAAAATCGCAATAGCAGC |
| prsA-R | TGCCAACTGTCGGAACGAGACTTCTTTATTTAGAATTGCTTGAAGATG |
| pDDXG-F3 | AGAAGTCTCGTTCCGACAGTTGG |
| pDDXG-R3 | ATTTCCCCCTTTGATTTAAGTGAAC |
| SRP-F | CAACCAACACCAATTAAAGGAGGAAATGGCATTTGAAGGATTAGCCGACC |
| SRP-R | AAGCCTGACTGGCGGTTAAATTGCCCCATTATACATGAACCTTGTTTCG |
| Ftsy-F2 | CAACCAACACCAATTAAAGGAGGAAATGAGCTTTTTTAAAAAATTAAAAG |
| Ftsy-R2 | AAGCCTGACTGGCGGTTAAATTGCTTAATCGTCGGCTTTTTCCACTAAATC |
| secA-F2 | CAACCAACACCAATTAAAGGAGGAAATGCTTGGAATTTTAAATAAAATG |
| secA-R2 | AAGCCTGACTGGCGGTTAAATTGCCTATTCAGTACGGCCGCAGC |
| secYEG-F | CAACCAACACCAATTAAAGGAGGAATTGTTTAAAACAATCTCCAAC |
| secYEG-R | AAGCCTGACTGGCGGTTAAATTGCCTATAGGATATAAGCAAGCGCAAT |
| secDF-F2 | CAACCAACACCAATTAAAGGAGGAAATGAAAAAAGGACGCTTGATTGC |
| secDF-R2 | AAGCCTGACTGGCGGTTAAATTGCTTATTGCGCCGAATCTTTTTTCAG |
| groESL-F2 | CAACCAACACCAATTAAAGGAGGAATTGTTAAAGCCATTAGGTGATCG |
| groESL-R2 | AAGCCTGACTGGCGGTTAAATTGCTTACATCATTCCACCCATACC |
| dnaK-F2 | CAACCAACACCAATTAAAGGAGGAAATGTCAGAAGAAAAACAAACC |
| dnaK-R2 | AAGCCTGACTGGCGGTTAAATTGCGGGTAATCATTGGGGTCGAGC |
| sipT-F2 | CAACCAACACCAATTAAAGGAGGAATTGACCGAGGAAAAAAATACGAATAC |
| sipT-R2 | AAGCCTGACTGGCGGTTAAATTGCTTATTTTGTTTGACGCATTTCG |
| sipS-F2 | CAACCAACACCAATTAAAGGAGGAATTGAAATCAGAAAATGTTTCGAAG |
| sipS-R2 | AAGCCTGACTGGCGGTTAAATTGCCTAATTTGTTTTGCGCATTTCG |
| pDD23-F | GCAATTTAACCGCCAGTCAGGCTTTC |
| pDD23-R | TTCCTCCTTTAATTGGTGTTGGTTGT |

**Additional Figure S1**

**The construction of all the integration plasmids used in this study**. **A**: the construction of pDDXG carrying the promoters P*_xylA_* and P*_grac_*. pDDXBG and pDDXGB were constructed to examine the effect of P*_xylA_* and P*_grac_*. **B**: the construction of 23 integration plasmids for single gene or gene operon overexpression. The 23 genes or gene operons were all under the control of the promoter P*_xylA_*, respectively. **C**: the construction of 9 integration plasmids for *prsA* overexpression combined with 9 genes or gene operons screened out, respectively. *prsA* was under the control of P*_xylA_*; the 9 genes or gene operons were under the control of P*_grac_*, respectively. The details of plasmid construction are described in Methods and Materials.
